# Thermal conductivity of poly(3,4-ethylenedioxythiophene) films engineered by oxidative chemical vapor deposition (oCVD)†

**DOI:** 10.1039/c8ra03302a

**Published:** 2018-05-25

**Authors:** Phil M. Smith, Laisuo Su, Wei Gong, Nathan Nakamura, B. Reeja-Jayan, Sheng Shen

**Affiliations:** Department of Mechanical Engineering, Carnegie Mellon University Pittsburgh PA 15213 USA bjayan@andrew.cmu.edu shengshe@andrew.cmu.edu

## Abstract

Oxidative chemical vapor deposition (oCVD) is a versatile technique that can simultaneously tailor properties (*e.g.*, electrical, thermal conductivity) and morphology of polymer films at the nanoscale. In this work, we report the thermal conductivity of nanoscale oCVD grown poly(3,4-ethylenedioxythiophene) (PEDOT) films for the first time. Measurements as low as 0.16 W m^−1^ K^−1^ are obtained at room temperature for PEDOT films with thicknesses ranging from 50–100 nm. These values are lower than those for solution processed PEDOT films doped with the solubilizing agent PSS (polystyrene sulfonate). The thermal conductivity of oCVD grown PEDOT films show no clear dependence on electrical conductivity, which ranges from 1 S cm^−1^ to 30 S cm^−1^. It is suspected that at these electrical conductivities, the electronic contribution to the thermal conductivity is extremely small and that phonon transport is dominant. Our findings suggest that CVD polymerization is a promising route towards engineering polymer films that combine low thermal conductivity with relatively high electrical conductivity values.

## Abstract 2

Measuring the thermal conductivity of oxidative chemical vapor deposited poly(3,4-ethylenedioxythiophene) thin films.

## Graphical abstract

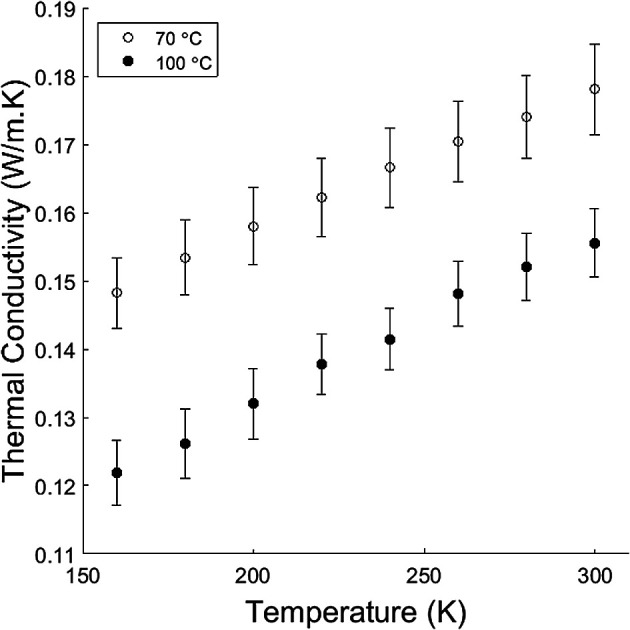

Molecular scale engineering tools like chemical vapor deposition (CVD) are the workhorses of the microfabrication industry. With these primarily inorganic thin film coating technologies, we can achieve multifunctional properties on the surface of a material which are different from those of the underlying substrate. CVD polymerization is a new technique that merges CVD thin film processing with the versatility of organic chemistry. This vapor phase polymerization offers a facile, solvent-free and low temperature route to simultaneously tune chemistry, morphology and functionality,^1^ allowing for creative ways to engineer multiscale (thicknesses from nano to micro) and multifunctional (insulating, semiconducting, conducting) polymer films on a variety of substrates including paper, plastic, and biological tissue.^1^

Oxidative chemical vapor deposition (oCVD) is the vapor phase equivalent of solution-based oxidative (step growth) polymerization. oCVD enables the polymerization of thin films of electrically conducting polymers such as poly(3,4-ethylenedioxythiophene) (PEDOT).^1,2^ As depicted in Fig. 1(a), the all-dry oCVD thin film polymerization process involves subliming and reacting a solid-state oxidant like iron(iii) chloride (FeCl_3_) with heated vapors of the EDOT monomer. Polymerization and thin film coating occurs simultaneously onto the surface of a temperature controlled substrate placed inside the custom-designed oCVD reactor. As we show in this work, electrical conductivity of the PEDOT films can be tailored (without affecting thermal conductivity) simply by varying the substrate temperature, synthesizing nanoscale PEDOT films whose properties can be precisely controlled in ways that are unattainable by solution processing.

**Fig. 1:**
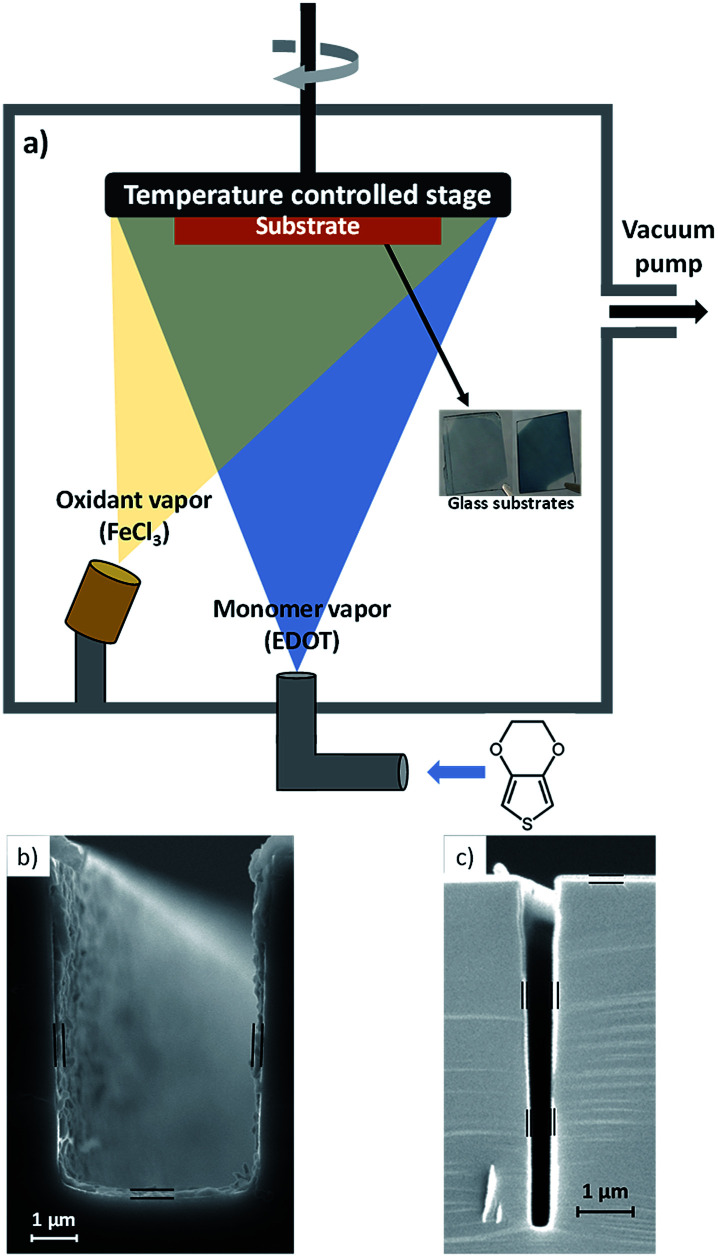
(a) Schematic of oCVD reactor. Inset shows two glass substrates coated with PEDOT films of different thicknesses. (b) & (c) Cross-sectional scanning electron microscopy of polymer coatings grown by CVD polymerization on silicon trenches.

Due to its high electrical conductivity, optical transparency, mechanical flexibility, and chemical and physical stability, PEDOT is one of the most widely studied conducting polymers.^3^ Commercially available PEDOT solutions are a mixture of PEDOT with the surfactant poly(styrenesulfonate) (PSS).^25^ This surfactant enables the dispersion of PEDOT in polar solvents, making processing techniques such as spin coating^4^ and screen printing^5^ a viable option for depositing PEDOT thin films. However, the highly polar PSS is strongly acidic and can cause failure in devices such as polymer solar cells.^6^ Although solution processing techniques may be widespread, realizing nanoscale film thicknesses will often lead to non-uniformity due to de-wetting and surface tension effects that accompany solution processes.

De-wetting and surface tension also make textured surfaces^7^ difficult to coat with the aqueous PEDOT:PSS while preserving the morphology of the structure underneath. On the other hand, CVD polymer films are uniform and pinhole free,^22^ exempt from de-wetting and surface tension effects. For this reason, extremely thin films are also attainable. In oCVD, reactants arrive at textured surfaces from all sides in the vapor phase, resulting in completely conformal PEDOT films that follow the contours of complex geometries.^8,9^ We show in Fig. 1(b) and (c) that CVD grown polymer films uniformly cover textured surfaces like trenches cut into silicon wafers, enabling potential applications in macro and micro scale device fabrication. This vapor phase process is further compatible with substrates which would dissolve or degrade in the presence of solvents. This enables the creation of devices on fragile materials such as paper^10^ which would lose its structural integrity if exposed to any type of solvent. Copolymerization is also possible, providing access to functional groups which are not inherent to the specific polymer of interest.^11^ The low cost,^23^ mechanical flexibility and varied functionality offered by CVD polymerization is thus unmatched by existing solution processing methods.

Herein, we use the differential 3ω method to measure the thermal conductivity of oCVD grown PEDOT films for the first time. In the oCVD process, by simply changing the substrate temperature, the conjugation length of the conducting polymer can be modified, thereby altering the electrical conductivity.^12^ We studied the thermal conductivity of films grown at different substrate temperatures to determine whether a correlation exists between thermal and electrical conductivity. The transmission line method (TLM) was used to measure the electrical conductivity.^16^

The oCVD process used to deposit PEDOT films is described in detail in ESI (S1†).^1,12^ Briefly, cleaned substrates were mounted onto a heating stage controlled from 70 °C to 130 °C. The monomer jar was heated to 130 °C while the oxidant was sublimed in a heated crucible at 225 °C. The deposition was carried out at a chamber pressure of 100 m torr for 45 min. After the deposition, the films were rinsed in methanol for 5 minutes to remove any residual monomer or oxidant. The resulting films had thicknesses ranging from 50–100 nm. The substrate used in this work was a silicon wafer with a thermally grown oxide layer. A 100 nm thick blocking layer of the electrically insulating polymer poly(divinylbenzene) (PDVB), was also deposited by CVD as described in the ESI S2.†^13^ This electrically insulating layer is necessary to accurately measure the thermal conductivity of electrically conductive materials using the 3ω method.

Performing temperature dependent measurements on soft, nanoscale organic layers like the oCVD grown polymer films is challenging. We describe our technique here in detail, particularly our device design and novel materials, such as the use of low temperature CVD grown PDVB blocking layer. The 3ω method used to measure the thermal conductivity of the oCVD PEDOT thin films is classified as a transient technique where a metal line acts simultaneously as a source heater and thermometer.^14^ When driven with a sinusoidal current at 2ω, the metal line heater causes temperature fluctuations in the sample also at 2ω. This in turn causes a small voltage signal across the heater at 3ω. The nature of the temperature fluctuation in the sample is dependent on the sample's thermal properties, including thermal conductivity. Specifically, the differential 3ω technique was chosen because (a) knowledge of the thermal properties of the substrate and other materials deposited on the substrate are not required, and (b) the error associated with the differential 3ω method has been shown to be less than that of the slope-based 3ω method for samples with multiple films.^14^Fig. 2 depicts the sample structure used here. Similarly, a reference sample identical to this sample except for the oCVD PEDOT film was also fabricated. It should be noted that the thermal conductivity obtained is the effective thermal conductivity because it includes interface thermal resistance.

**Fig. 2:**
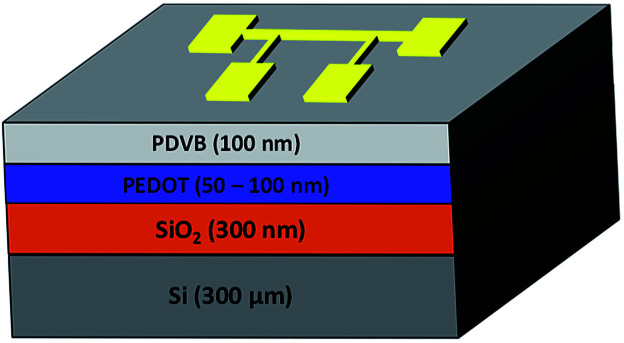
Device structure for 3ω measurement.

A stainless-steel mask was used in conjunction with electron beam (e-beam) evaporation to fabricate the gold line heater on top of the PDVB layer. Metal lines with widths ranging from 40 μm to 70 μm were obtained. Since PEDOT is electrically conducting, the PDVB layer served to isolate the PEDOT film from the metal line heater, preventing current leakage and thus error in the measurement. Typically, processes such as sputtering or physical vapor deposition are used to deposit an electrically insulating layer such as silicon dioxide, however, sputtering may damage the polymer film and the high temperature involved will also be detrimental. In contrast, CVD polymerization of PDVB requires low substrate temperature (20 °C to 70 °C) and does not damage the PEDOT layer. The same mask and procedure was used to fabricate the line heaters on the corresponding reference samples as well.

A wedge wire bonder was used to make electrical connections between the contact pads of the chip carrier and the contact pads of the line heater. However, due to the delicate and soft nature of PEDOT films, directly bonding to the line heater contact pads resulted in film damage and bond detachment. To facilitate the wire bonding process, a technique similar to the one described by Kaul *et al.*^15^ was used. A small amount of conductive epoxy was first applied to each contact pad of the gold line heater. After curing, wire bonding was then done directly from the solid epoxy to the pads on the chip carrier. This epoxy bonding technique was also utilized when measuring the electrical conductivity of the PEDOT films using TLM (Fig. 3 inset).^16^

**Fig. 3:**
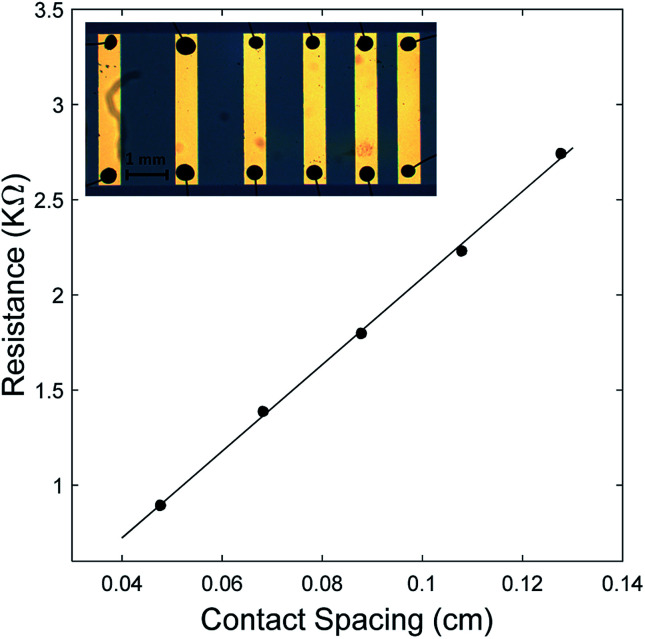
Resistance *vs.* contact spacing of oCVD PEDOT film grown at a substrate temperature of 100 °C. Inset is an optical image of metal contacts with different spacing between pairs of electrodes.

For the TLM measurements, a stainless-steel mask was first used to pattern the PEDOT film on the substrate. Gold electrodes with varying spacing between pairs of electrodes were then deposited directly onto the PEDOT film, using another stainless-steel mask and e-beam evaporation. 4-Point resistance measurements were done between each pair of electrodes to obtain a plot of resistance *versus* length. Fig. 3 shows results for the sample deposited at 100 °C, where the measured resistance is plotted as a function of distance between electrodes. The electrical conductivity is then inferred from the slope of the linear graph. The inset in Fig. 3 shows the gold electrodes with varying distances between pairs of electrodes on the PEDOT film. The conductive epoxy and bonded wires are also shown. The data from other samples measured were similar to that of Fig. 3.

Table 1 summarizes thermal and electrical conductivity values measured at room temperature for PEDOT films deposited at 70 °C, 100 °C and 130 °C, with 130 °C being the upper limit of the oCVD reactor. These three temperature values were chosen in order to observe a significant and measurable change in electrical conductivity. From the data, a clear correlation between electrical conductivity and substrate temperature is observed. This behaviour is due to an increase in the conjugation length of the PEDOT film with elevated substrate temperature, which can be directly correlated to an increase in electrical conductivity.^12^ Comparing the films deposited at 70 °C and 100 °C, we observe an order of magnitude increase in the electrical conductivity. However, a further increase in the substrate temperature to 130 °C only results in a 20% increase in the film's electrical conductivity. One explanation for this result is the formation of hydrogen chloride (HCl) during the polymerization.^12^ During the step growth polymerization, the oxidant initiates the reaction with the monomer to generate a radical cation, the deprotonation of the carbon–carbon coupled monomers generate the acidic HCl which acts as an inhibitor, and reduces the film's electrical conductivity. As the substrate temperature increases the HCl content is reduced due to evaporation, resulting in a higher electrically conductive film.^24^ Nevertheless, it appears that a saturation point was attained in our system, after which, an increase in temperature will no longer cause the HCl to evaporate. This effect could potentially explain why only a modest increase in electrical conductivity is observed for a substrate temperature increase from 100 °C to 130 °C. From Table 1, we also see that there is no relation between thermal conductivity and film thickness. Since amorphous polymers have a small phonon mean free path (<10 nm),^17^ phonon boundary scattering effects are not expected since the thickness of the films in this study is >10 nm.

**Table 1:** Thermal and electrical conductivities of oCVD PEDOT films deposited at different substrate temperatures. Both quantities were measured at room temperature

Substrate temperature [°C]	70	100	130
Film thickness [nm]	95	64	53
Thermal conductivity [W m^−1^ K^−1^]	0.185	0.156	0.319
Electrical conductivity [S cm^−1^]	1.79	18.40	22.26

Fig. 4 shows that the thermal conductivity of oCVD grown PEDOT films increases with temperature. A similar behavior has also been observed from the conducting polymer polyaniline within the same temperature range.^15^ This comes as a result of the thermal conductivity's dependence on heat capacity in the measured temperature range.^17^ The temperature range was restricted to 160–300 K due to experimental constraints. The uncertainty in the measurements was calculated using the Kline McClintock method.^18^ The inset in Fig. 4 is an optical image of the line heater used in the 3ω method for measuring the thermal conductivity. Similar trends were observed for the samples deposited at 70 °C and 130 °C (ESI Fig. S4†).

**Fig. 4:**
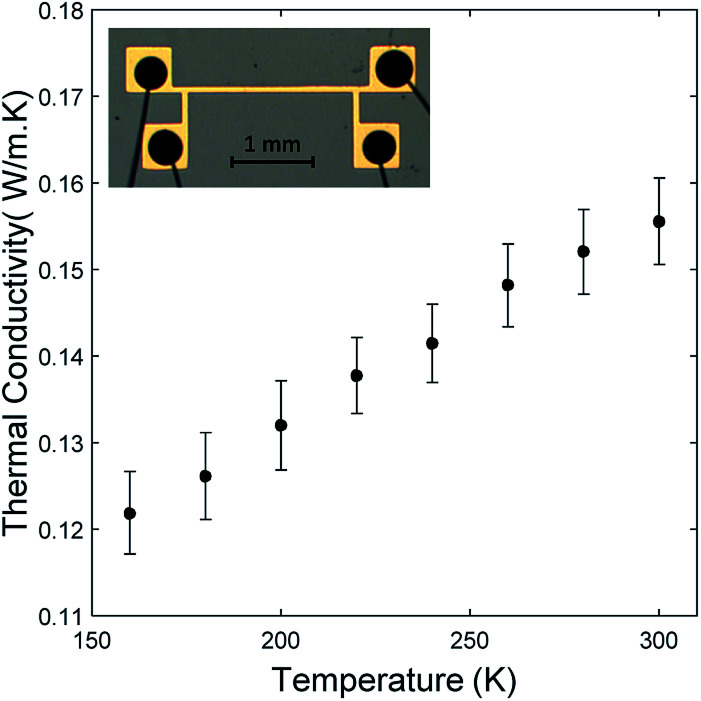
Temperature dependent thermal conductivity of oCVD PEDOT film grown at a substrate temperature of 100 °C. Inset is an optical image of the line heater with conductive epoxy and bonded wires.

From the room temperature values shown in Table 1, no clear correlation between thermal and electrical conductivity can be deduced at these electrical conductivity values. The thermal conductivity of solution processed PEDOT:PSS typically falls within the ranges of 0.3 to 2.2 W m^−1^ K^−1^.^19,20^ However, as seen in Table 1, oCVD PEDOT films can attain lower thermal conductivities at room temperature. One possibility is due to the absence of PSS. A large fraction of the covalent bonds present in PEDOT:PSS is contributed by the relatively large PSS molecule. Compared to van der Waals bonds, these stiff covalent bonds are more effective at transferring thermal energy between polymer chains.^21^ This suggests that PEDOT films synthesized without PSS could have predominately van der Waals bonds allowing for lower thermal conductivity.

## Conclusions

The goal of this study is to conduct benchmark tests of thermal conductivity for oCVD PEDOT thin films. The thermal conductivity of films deposited at different substrate temperatures were measured. Room temperature thermal conductivity values as low as 16 W m^−1^ K^−1^ are obtained using the differential 3ω method. Although a clear dependence on substrate temperature is reflected in the electrical conductivity, no clear correlation between electrical and thermal conductivity is seen. Since electrons and phonons are heat carriers, the thermal conductivity can have both electronic and phonon contributions. Hence, an increase in electrical conductivity and thus electrons would result in an increase in thermal conductivity. From our work, considering the films deposited at a substrate temperature of 70 °C and 100 °C, the electrical conductivity increases by a factor of ∼10 however, the thermal conductivity decreased by a factor of ∼1.2. For the films deposited at a substrate temperature of 100 °C and 130 °C, the electrical conductivity increases by a factor of ∼1.2 however, the thermal conductivity increased by a factor of ∼2. The inconsistencies in these results lead us to believe that the electronic contribution to the thermal conductivity is low and that phonon transport is dominant. Our thermal measurements thus provide evidence that CVD polymerization is a promising new approach to engineer PEDOT thin-films which combine low thermal conductivity with relatively high electrical conductivity values. Furthermore, the highly tunable nature of this CVD technique opens up opportunities for precisely tailoring the chemical composition, morphology, and electrical and thermal conductivities of polymer films to meet specific device/application requirements.^1^ This will be beneficial to many areas such as thermoelectric generators, photovoltaics, and lithium ion batteries.

## Conflicts of interest

There are no conflicts of interest to declare.

## Supplementary Material

RA-008-C8RA03302A-s001
