# Supplementary material for: Thermal conductivity of poly(3,4-ethylenedioxythiophene) films engineered by oxidative chemical vapor deposition (oCVD)
Source: RSC Adv. 2018 May 25;8(35):19348–52. doi: 10.1039/c8ra03302a (PMC9080664; doi:10.1039/c8ra03302a)
Supplement: RA-008-C8RA03302A-s001 [file RA-008-C8RA03302A-s001.pdf]

Electronic Supplementary Material (ESI) for RSC Advances.
This journal is © The Royal Society of Chemistry 2018

Supplementary Information

Thermal Conductivity of Poly (3,4-ethylenedioxythiophene) Films Engineered by oxidative Chemical Vapor Deposition (oCVD)

Phil M Smith[a], Laisuo Su[a], Wei Gong[a], Nathan Nakamura[a], B. Reeja-Jayan[a] and Sheng Shen[a]

[a] *Department of Mechanical Engineering, Carnegie Mellon University, Pittsburgh PA 15213*

S1. Oxidative Chemical Vapor Deposition (oCVD) of PEDOT films

The monomer EDOT was purchased from Sigma-Aldrich and used without further purification. The deposition conditions used are summarized in Table S1. With the exception of the substrate temperature, all parameters listed in table S1 were optimized for control over the deposition rate. The argon gas was used to maintain the process pressure and the substrate temperature was used to tune the electrical conductivity of the film. The oCVD procedure has been previously report by Im et al.[1] Briefly, the monomer (EDOT) was placed in a stainless-steel jar and heated to 130 ˚C. The samples were placed on a heated rotating stage maintained at temperatures outlined in Table S1. The EDOT and Argon were introduced into the chamber at 2 sccm and 1 sccm respectively and the oxidant was sublimed at 225 ˚C. The depositions were conducted at a pressure of 100 mTorr for 45 mins. The samples were then rinsed in methanol for 5 mins before further measurements were conducted.

The thickness of the oCVD PEDOT films were measured using a KLA Tencor P-15 profilometer. The film was scratched in multiple locations to expose the underlying substrate such that the film thickness can be measured. For this, a soft tip tweezer was used to avoid placing scratches in the substrate itself. The thickness was measured at multiple locations across the sample and then averaged. In a separate set of experiments, the uniformity of the oCVD PEDOT films were measured using ellipsometry, results are shown in figure S1 and table S2. The FTIR spectrum of the oCVD film is shown in figure S2. The C=C asymmetric stretching mode is seen at 1516 cm^{-1}, the C-C inter-ring stretching mode is seen at 1317 cm^{-1}, the peaks appearing from 1188 cm^{-1} to 1053 cm^{-1} corresponds to the C-O-C bending vibration in ethylenedioxy group and the peaks appearing from 977 cm^{-1} to 837 cm^{-1} corresponds to the C-S-C stretching vibration in the thiophene ring.

Table S1. Summary of oCVD PEDOT parameters

Substrate temperature (˚C)	70	100	130
Monomer flow rate (sccm)	2	2	2
Argon (sccm)	1	1	1
Chamber pressure (mTorr)	100	100	100
Evaporator temperature (˚C)	225	225	225
Deposition time (mins)	45	45	45

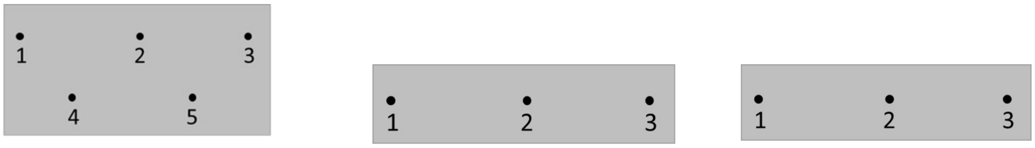

Figure S1. PEDOT thin films on Si wafer for different coating time. (left) 5 mins, (middle) 10 mins, (right) 20 mins. The points with number beside them are positions during ellipsometry measurement.

Table S2. PEDOT Film thickness measured by ellipsometry at different locations for three different samples, as shown in Figure S1.

Point		1	2	3	4	5
Thickness (nm)	Sample 1	13.9	15.9	13.8	15.1	15.6
	Sample 2	22.9	22.3	22	/	/
	Sample 3	49.3	48.4	48.6	/	/

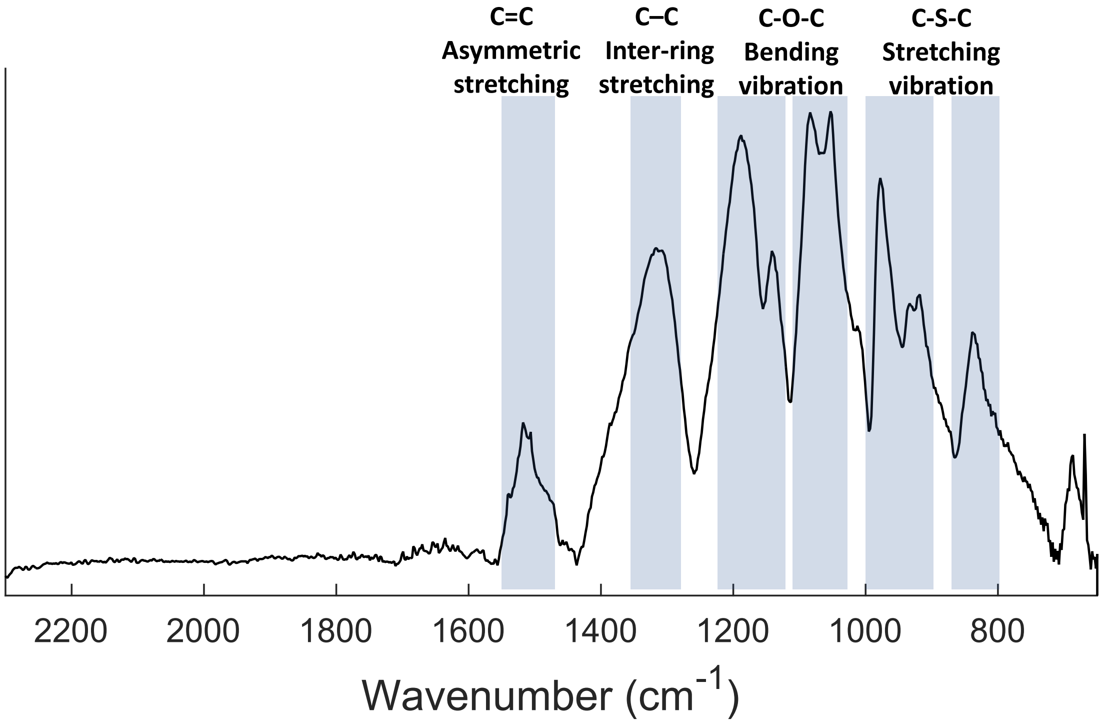

Figure S2. FTIR spectrum of oCVD PEDOT films.

S2. Initiated Chemical Vapor Deposition (iCVD) of PDVB films

The monomer divinylbenzene (DVB) and initiator TBPO were purchased from Sigma-Aldrich and used without further purification. Details about the iCVD polymerization of poly(divinylbenzene) (PDVB) have been reported previously[2], and the deposition conditions are summarized in Table S3. Briefly, DVB was heated in a stainless-steel jar at 65 °C and its vapors along with vapors of tert-butyl peroxide (TBPO) (at room temperature) were delivered into the reactor at flow rates of 2.0 sccm and 1.3 sccm, respectively. Argon gas was also introduced into the reactor at 8.5 sccm. The labile peroxide bond of the initiator was thermally cleaved by resistively heated nichrome filaments inside the CVD reactor to produce free radicals that attack vinyl bonds on DVB and initiate free-radical polymerization. iCVD is a substrate-independent process as the substrate temperature remains close to room temperature. Here, the reactor pressure, substrate temperature, and filament temperature were maintained at 500 mTorr, 25 °C, 230 °C, respectively. A schematic of the iCVD reactor is shown in Figure S4.

Table S3. Summary of iCVD PDVB parameters

Parameter	Values
Substrate temperature (°C)	25
Filament temperature (°C)	230
Reactor pressure (mTorr)	500
Initiator flow rate (sccm)	2.0
Monomer flow rate (sccm)	1.3
Argon flow rate (sccm)	8.5
* P_m/P_{sat}	0.14

* P_m is the partial pressure of monomer inside chamber, P_{sat} is the saturation pressure of monomer on the substrate. P_m/P_{sat} can be viewed as a direct measure of monomer surface concentration on the substrate.

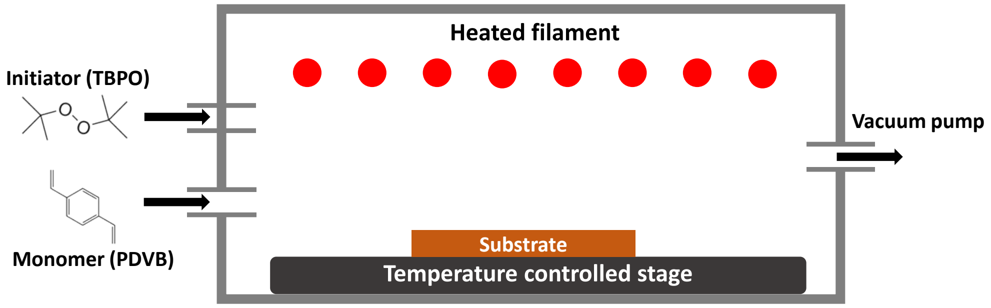

Figure S3. Schematic of iCVD reactor. A heated jar is used to introduce the monomer vapor into the reactor. The volatile initiator is introduced at room temperature and decomposes into free radical by the heated filament. These free radicals initiate free-radical polymerization with the vinyl bonds on DVB.

S3. Scanning Electron Microscopy (SEM) Results

Cross-sectional scanning electron microscopy (SEM) was utilized to check conformality and verify thickness measurements for PEDOT films. Sample cross sections were prepared by cutting samples using a diamond tipped pen. Images were recorded using an FEI Quanta 600 FEG SEM operating in secondary electron mode, with an accelerating voltage of 10 kV and a spot size of 3. An image of a plain trench without polymer coating is shown in Figure S2.

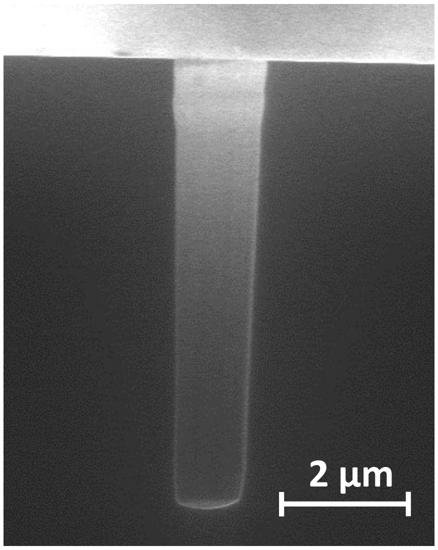

Figure S4. Cross-sectional SEM of a plain trench without polymer coating

S4. Cross-plane thermal conductivity using the differential 3ω Method

When measuring the thermal conductivity of a thin film deposited on a thick substrate, if the film thickness is much smaller than the width of the line heater, the component of the temperature fluctuation caused by the film (ΔT_f) is frequency independent. As such the temperature fluctuation across the sample is given by,

$$\Delta T_{Total}(\omega) = \Delta T_{Sub}(\omega) + \sum_{i=1}^{n} \Delta T_i , \qquad (1)$$

where ω is the angular frequency, ΔT_{Sub} is the component of ΔT_{Total} caused by the substrate, ΔT_i is the component of ΔT_{Total} caused by the *i*-th film, and n is the total number of films on the substrate. By creating a reference sample identical to the sample of interest, with the exception of the film of interest, the difference between the temperature fluctuations of the two samples will be solely due to the presence of the film of interest. As such, the component of the temperature fluctuation caused by the film of interest is simply

$$\Delta T_f = \Delta T_{Total}(\omega) - \Delta T_{ref}(\omega), \qquad (2)$$

where ΔT_{ref} is the temperature fluctuation from the reference sample. Once the component of the temperature fluctuation caused by the film is measured, its thermal conductivity is given by,

$$k_f = \frac{P\, t_f}{L\, W\, \Delta T_f} , \qquad (3)$$

where k_f and t_f are the thermal conductivity and thickness of the film of interest and P, L and W are the power, length and width of the line heater, respectively.

The stainless-steel mask was made using a 75 µm thick stainless-steel sheet. A LPKF Protolaser U3 was used to cut the sheet into the desired pattern necessary for performing 3ω and transmission line measurements. A Westbond 7476D wedge wire bonder was used to make contact between the

sample and the DIP chip carrier. Temperature dependent measurements were performed in a Jantis ST-500 cryostat cooled with liquid nitrogen with the samples mounted onto a 24-pin DIP chip carrier. The experimental setup is shown in Figure S3. The temperature dependent thermal conductivity results for the films deposited at 70 ˚C, 100 ˚C and 130 ˚C are shown in Figure S4.

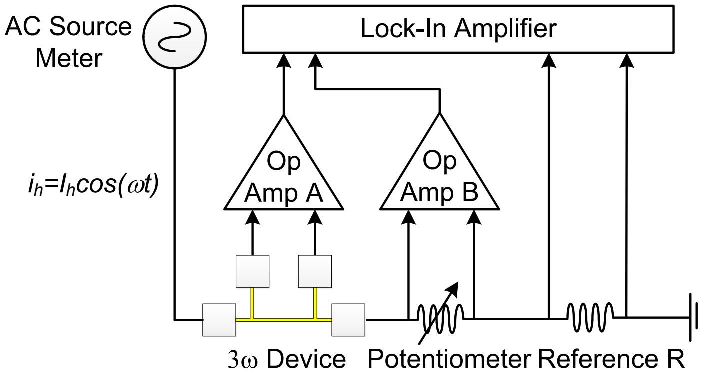

Figure S5. Schematic of experimental setup for 3ω method. A Keithley 6200 source meter served as the AC source. The potentiometer was used to match the 1ω signal from the 3ω device. This allowed the 3ω signal to be isolated by the lock-in amplifier.

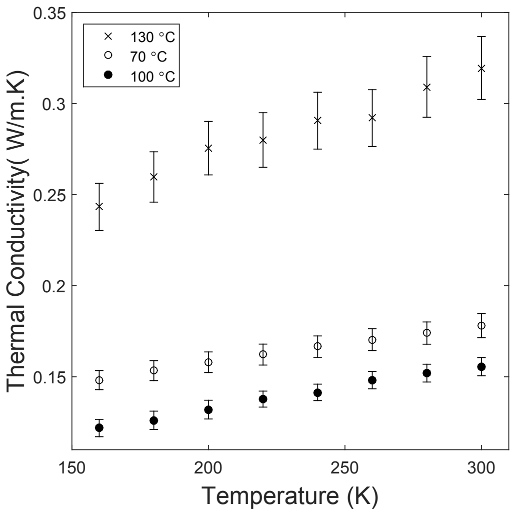

Figure S6. Temperature dependent thermal conductivity of oCVD PEDOT grown at substrate temperatures of 70 ˚C, 100 ˚C and 130 ˚C.

S5. Transmission Line Method (TLM)

The total resistance (R_T) measured across two electrodes is given by:

$$R_T = \frac{R_s L}{W} + 2R_c \quad (4)$$

where R_s is the sheet resistance, L is distance between electrodes, W is the width of the film and R_c is the contact resistance. Plotting R_T Vs L, R_s/W can be inferred from the slope (m) of a linear fit. Since

$$R_s = \frac{\rho}{t} = \frac{1}{t\,\sigma} \tag{5}$$

where t is the film thickness, ρ is the sheet resistivity and σ is the electrical conductivity, from (4) and (5)

$$\sigma = \frac{1}{t\,W\,m} \tag{6}$$

Reference

[1]Q. Zhao, R. Jamal, L. Zhang, M. Wang and T. Abdiryim, Nanoscale Res. Let., 2014, **9**, 557.

[2]C. D. Petruczok, R. Yang and K. K. Gleason, Macromolecules, 2013, **46**, 1832-1840.
